# Expression of the metastasis suppressor BRMS1 in uveal melanoma

**DOI:** 10.3332/ecancer.2014.410

**Published:** 2014-03-11

**Authors:** Bruna V Ventura, Carlos Quezada, Shawn C Maloney, Bruno F Fernandes, Emilia Antecka, Claudia Martins, Silvin Bakalian, Sebastian di Cesare, Miguel N Burnier

**Affiliations:** Department of Ophthalmology and Pathology, The Henry C. Witelson Ocular Pathology Laboratory, McGill University, Montreal, Quebec H3A 2B4, Canada

**Keywords:** uveal melanoma, metastasis suppressor gene, BRMS1, metastasis

## Abstract

**Aims:**

To determine the expression of breast metastasis suppressor 1 (BRMS1) in human uveal melanoma (UM) tissues and cell lines. In addition, we intend to establish a possible association between BRMS1 expression and the presence of metastatic disease.

**Methods:**

Thirty-one formalin-fixed paraffin-embedded tissues from enucleated eyes of patients with UM were immunostained. Clinical–pathological data were obtained, including age, tumour location, largest dimension, cell type, and occurrence of metastasis. The expression of BRMS1 mRNA in four human UM cell lines was determined by real-time reverse transcriptase polymerase chain reaction, and protein expression was assessed by immunocytochemistry and western blot. The association between BRMS1 immunostaining and location, largest tumour dimension, and tumour cell type was determined using the correlation coefficient test. The association between BRMS1 immunostaining and the incidence of metastasis was assessed using Kaplan–Meier analysis.

**Results:**

Of the 31 cases of UM, 24 (77.42%) stained positive and seven (22.58%) negative for BRMS1. From the positively stained tumours, 21 (87.50%) showed cytoplasmatic staining. Macrophages were usually positive when present in the tumour and staining intensity was generally higher than in UM cells. BRMS1 mRNA was present in all four human UM cell lines, as well as cytoplasmatic immunoexpression of BRMS1. Immunoblotting showed variable BRMS1 protein levels between the different cell lines. No statistically significant correlation was found between BRMS1 protein expression and survival (*P* = 0.69), tumour cell type (*P* = 0.68), largest tumour dimension (*P* = 0.75), and tumour location (*P* = 0.11).

**Conclusions:**

BRMS1 is expressed in UM both at the mRNA and protein level; however, neither was associated with any of the prognosticor outcome parameters that we tested.

## Introduction

Uveal melanoma (UM) represents approximately 5% of all reported melanomas [1], with an incidence of five to seven cases per million people per year [2]. UM is the most common primary intraocular malignancy in adults. Despite improvements in diagnosis and treatment of the primary tumour [3–7], the survival rate of patients has remained relatively unchanged for the past few decades [8, 9]. The leading cause of death from this disease is metastasis [10, 11]; it is estimated that 40–50% of patients with UM [12] develop metastasis within ten years of the initial diagnosis [13, 14]. The median survival time from the identification of metastasis varies, depending on the study, from less than six to up to 12.5 months [14–16].

The traditional concept that metastasis occurs during late stages of tumourigenesis [10] has been replaced by evidence showing early tumour cell dissemination and metastatic disease developing in parallel with the primary tumour [17]. Callejo *et al* [18] recorded the presence of circulating malignant cells in patients with UM at the time of diagnosis despite the size of the primary tumour, indicating that UM is a cancer that metastasizes early.

The metastatic cascade involves complex, interrelated, and essential steps [19]. Genes that regulate metastasis are classified as either metastasis-promoting or metastasis-suppressing genes [20]. Metastasis-promoting genes drive conversion of tumours from non-metastatic to metastatic, while metastasis-suppressing genes block metastasis without affecting tumourigenicity [20]. The first metastasis suppressor gene (MSG) described was NM23. Ma *et al* [21] have shown that NM23 mRNA and protein expression are closely correlated with reduced metastatic behaviour in a UM animal model. Previously published work showed that NM23 mRNA expression is associated with lower metastatic potential of human UM cell lines, while high immunostaining intensity in patient samples is associated with better survival [22]. Another study, involving the MSG *KISS1*, revealed a strong correlation between KISS1 immunoreactivity and lower risk of metastatic disease in UM and a better survival rate [23]. To date, these are the only MSGs studied in UM.

Breast metastasis suppressor 1 (BRMS1) is a gene that maps to chromosome 11q13.1–q13.2 [20, 24] and is translated into a 246-amino acid-long, predominantly nuclear protein [20]. A previous study has shown a dose-dependent significant decrease in the metastatic potential of BRMS1-transfected breast carcinoma cells in an animal model, while no change in tumourigenicity was observed [20]. Shevde *et al* [25] have correlated a decrease in BRMS1 mRNA levels with an increase in the metastatic potential of skin melanoma cell lines. The results of other studies suggest that BRMS1 also suppresses metastasis in ovarian carcinoma and human bladder cancers [26, 27]. Since the survival rate in UM patients has remained unchanged and metastasis develops in almost half of them and is the leading cause of death, it is important to conduct research that will result in better understanding of the disease, and to find markers that may serve as predictors of more aggressive metastatic disease or even as potential therapeutic factors.

The metastasis suppressor BRMS1 has not been studied in UM. Therefore, our purpose is to investigate the immunohistochemical expression of BRMS1 in human UM specimens, and to establish if there is an association between its expression and metastatic disease. In addition, we aim to determine BRMS1 mRNA and protein expression in human UM cell lines.

## Materials and methods

### Tissue samples

Thirty-one formalin-fixed paraffin-embedded blocks of enucleated primary tumours from patients with UM were collected from the archives of the Henry C. Witelson Ocular Pathology Laboratory and Registry, McGill University, Montreal, Canada.

Tumour cell type, as recorded in the original pathology report, was reclassified according to the modified Callender’s classification of UM [30]. Tumours composed of only one type of cell were classified as epithelioid or spindle, according to the cell type. Tumours containing both spindle and epithelioid cells were classified as mixed. The patient’s medical charts and cancer registry were reviewed to provide age at diagnosis, tumour location, largest tumour dimension, tumour cell type, and occurrence of metastasis.

### Immunohistochemistry

Formalin-fixed paraffin-embedded sections of the collected blocks were stained with haematoxylin and eosin for histopathological assessment.

Immunohistochemistry was performed using the Ventana BenchMark LT fully automated machine (Ventana Medical System Inc, Arizona, United States). The fully automated processing of bar code labelled slides included baking of the slides, solvent-free deparaffinisation, and CC2 (Citrate buffer pH 6.0) antigen retrieval. Slides were incubated with the mouse antihuman monoclonal antibody against BRMS1 (M01), clone 2D4-2G11 (Abnova Corporation, Taiwan), at a dilution of 1:500 for 40 min at room temperature, followed by application of biotinylated secondary antibody (8 min, 37 oC), then an avidin/streptavidin enzyme conjugate complex (8 min, 37 oC). Finally, the antibody was detected by Fast Red chromogenic substrate and counterstained with haematoxylin.

As a positive control, sections of breast cancer were used, as this cancer has been shown to express BRMS1 protein [20]. For negative control, the primary antibody was omitted.

### Microscopic classification

A single experienced pathologist blinded to the patients’ follow-up data analysed the stained sections. The sections were classified as group 1 when more than 30% of the neoplastic cells stained for BRMS1 and as group 2 when less than 30% stained. Both groups were subclassified into cytoplasmatic, nuclear, or nuclear–cytoplasmatic staining and into diffuse or focal staining.

### Cell lines

Four human UM cell lines (92.1, SP6.5, MKT-BR, and OCM-1) were incubated at 37 ºC in a humidified 5% CO2-enriched atmosphere. The cells were cultured in RPMI-1640 medium (Invitrogen, Burlington, Ontario, Canada), supplemented with 5% heat inactivated foetal bovine serum (Invitrogen), 1% fungizone (Invitrogen), and 1% penicillin–streptomycin (Invitrogen). Cells were cultured as a monolayer in 25-cm2 flasks (Fisher, Whitby, Ontario, Canada) and observed twice weekly, at every media change, for normal growth by phase contrast microscopy. The cultures were grown to confluence and passaged by treatment with 0.05% trypsin in ethylenediaminetetraacetic acid (EDTA; Fisher) at 37 ºC and washed in 7 mL RPMI-1640 media before being centrifuged at 120*g* for 10 min to form a pellet. Cells were then suspended in 1 mL of medium and counted in trypan blue using a haemocytometer.

The UM cell lines 92.1, SP6.5, MKT-BR, and OCM-1 were established by Dr. Jager (University Hospital Leiden, The Netherlands), Dr. Pelletier (Laval University, Quebec, Canada), Dr. Belkhou (CJF INSERM, France), and Dr. Albert (University of Wisconsin-Madison, United States), respectively. The characteristics of these cell lines are described elsewhere, and their metastatic potential (92.1 > SP6.5 > OCM-1 > MKT-BR) was determined using an immunosuppressed rabbit model [28] and later confirmed with *in vitro* studies [29].

### Quantitative real-time reverse transcriptase polymerase chain reaction

Total cellular RNA was extracted from the four human UM cell lines using the Qiagen RNeasy kit following the manufacturer’s instructions. Briefly, the cells were disrupted and homogenised using the included lysate buffer and extracted with a 20.5 gauge syringe. The lysate was then centrifuged to remove any insoluble material. One volume of 70% ethanol was added to the lysate and mixed, before the solution was added to the included RNeasy mini column. Following centrifuging, the columns were washed twice using the included buffer solutions. Total cellular RNA was then eluted using RNase free water.

Expression level of BRMS1 mRNA was subsequently determined by quantitative real-time reverse transcriptase polymerase chain reaction (RT-PCR) performed in triplicate using Quantitect one-step SYBR Green PCR method (Qiagen, Mississauga, Ontario, Canada) following the manufacturer’s instructions. A Chromo4 thermocycler (MJ Research) was used for all experiments and the results were analysed using the GeneEx software. QuantiTect primer assay pairs (Qiagen) for BRMS1. The β-actin housekeeping gene was also used in all experiments for the purpose of normalisation.

### Immunocytochemistry

Cytospins of four human UM cell lines with different metastatic potentials (92.1, SP6.5, OCM-1, and MKT-BR) were prepared using Cytospin3 centrifuge (Shandon Scientific Ltd, Pennsylvania, United States). Cells from culture were diluted to a concentration of 1 × 10^6^ cells/mL, and 300 μL was placed in each spin to be evenly plated on each slide. The spins were fixed with 2% paraformaldehyde. All slides were then immunostained with a mouse anti-human monoclonal antibody against BRMS1 (M01), clone 2D4-2G11 (Abnova Corporation), diluted 1:100, using the Ventana BenchMark LT fully automated machine (Ventana Medical System Inc) programmed to use a standard Avidin–Biotin Complex method.

### Western blot

Cells from the four human UM cell lines were lysed on ice in TNESV (50-mM TRIS HCL, 1% Nonidet P-40, 2-mM EDTA, 100-mM NaCl, 1-mM Orthovanadate) containing a protease inhibitor cocktail (Sigma) and the lysate was cleared by centrifugation for 10 min at 12000*g*. Protein concentration was determined by Bio-Rad protein assay (Bio-Rad Laboratories, Mississauga, Ontario, Canada). Protein aliquots of 10 μg were loaded onto 12% SDS-Page gel and then transferred to polyvinylidene fluoride-Millipore (PVDF) membrane.

The membrane was blotted for a mouse anti-human monoclonal antibody against BRMS1 (M01), clone 2D4-2G11 (~26.8 kDa; Abnova Corporation), at a dilution of 1:1000. Horseradish peroxidase conjugated goat anti-mouse secondary antibody (Santa Cruz Biotechnology, California, United States) was applied. Detection was performed using enhanced chemiluminescence with Pierce SuperSignal West Femto Maximum Sensitivity Substrate (Pierce Biotechnology Inc, Rockford, Illinois, United States) and captured with autoradiographic film (Hyperfilm-ECL; GE Healthcare, California, United States). β-actin was used for loading controls.

### Statistical analysis

Statistical analysis was performed using a computer-based statistical package (MedCalc Statistical Package, Version 9.2.0.2, Mariakerke, Belgium). The association between BRMS1 immunostaining and tumour location, largest tumour dimension, and tumour cell type was determined using the correlation coefficient test. The association between BRMS1 immunostaining and the incidence of metastasis was assessed using the Kaplan–Meier survival analysis. A *P*-value of less than 0.05 was considered statistically significant. All data accumulation was in accordance with country and provincial laws, and the tenets of the Declaration of Helsinki.

## Results

### UM patients

Thirty-one patients were included in our study. The mean age at the time of primary tumour diagnosis of UM patients was 61.13 years ± 12.61 (mean ± standard deviation) in both groups. In Table 1, we can see the age, location, histological classification, and tumour dimensions according to group. The number of patients who developed metastasis was 8/24 in group 1 and 2/7 in group 2.

### BRMS1 mRNA and protein expression in UM cell lines

**Figure 1.**
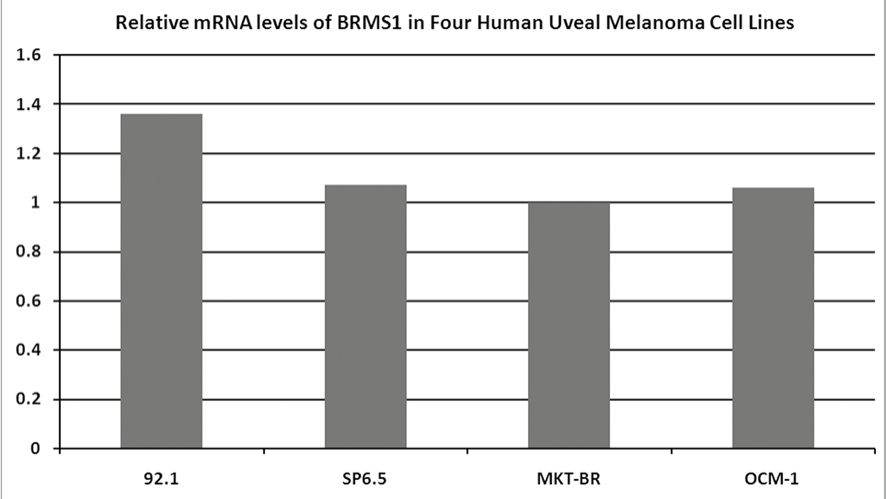
Expression levels of BRMS1 mRNA in four human UM cell lines, determined by RT-PCR. All four cell lines express BRMS1 in the mRNA level. The relative expression across cell lines is similar.

The RT-PCR showed that the BRMS1 mRNA was present in the four human UM cell lines. The relative expression across the cell lines was similar (Figure 1).

Immunoexpression of BRMS1 was evident in all four UM cell lines to a similar degree, independent of their metastatic potentials with a cytoplasmic staining pattern.

**Figure 2.**
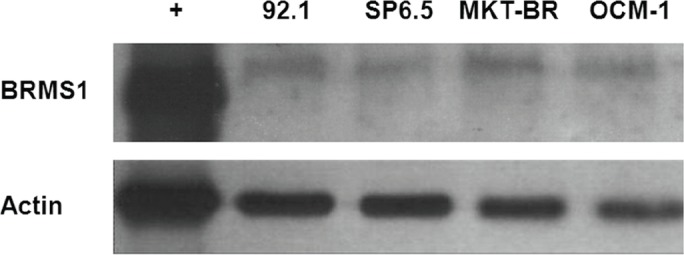
Western blot results revealing BRMS1 protein expression in four human UM cell lines. Immunoblotting shows a positive band at ~26.8 kDa for all cell lines. The cell lines had different BRMS1 protein levels; MKT-BR, the least metastatic of the four cell lines, had the highest protein expression.

Immunoblotting showed a positive band at ~26.8 kDa for all cell lines, with variable BRMS1 protein levels between the different cell lines. MKT-BR, the least metastatic of the four assayed cell lines, had the highest protein expression, and SP6.5, the second-most metastatic cell line, had the lowest protein expression (Figure 2).

### BRMS1 protein expression in samples of UM patients

**Figure 3.**
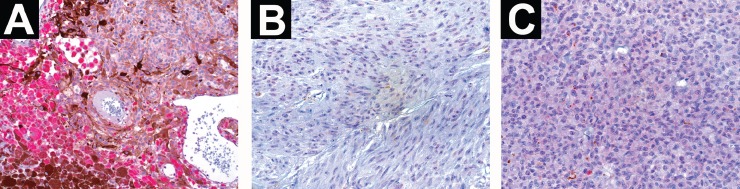
The immunostaining pattern of BRMS1 in primary human UM specimens. (a) UM specimen showing cytoplasmatic staining for anti-BRMS1. Macrophages at the lower left of the picture have a higher staining intensity than UM cells (×200 magnification). (b) UM specimen with nuclear staining (×400 magnification). (c) UM specimen demonstrating a nuclear–cytoplasmatic staining pattern (×400 magnification).

Of the UM cases studied, 24/31 were positive and 7/31 were negative for anti-BRMS1, forming, respectively, groups 1 and 2. From group 1, 21 of the tumours demonstrated cytoplasmatic staining, two had nuclear–cytoplasmatic staining, and one had nuclear staining (Figure 3); two of the tumours were focally stained while 22 were diffusely stained. Our breast cancer positive control specimen showed diffuse nuclear staining. Macrophages were generally positive when present in the tumour and their staining intensity was frequently higher than in UM cells. No statistically significant correlation was established between BRMS1 protein expression and survival (*P* = 0.69), tumour cell type (*P* = 0.68), largest tumour dimension (*P* = 0.75), or tumour location (*P* = 0.11).

## Discussion

For the first time, we have shown that BRMS1 is expressed in human UM tissue as well as in UM cell lines.

It is known that the metastatic cascade involves the coordinated expression of multiple genes to result in an established metastatic nodule, while it can only take one gene to block this process [24]. Since their discovery, MSGs have been viewed as possible prognostic indicators and therapeutic targets [31].

The only MSGs previously described in UM are NM23 and *KISS1* [21–23]. BRMS1 is an MSG first described in human breast cancer and it has been correlated with decreased metastatic disease in many other tumours [20, 25–27].

BRMS1 was first analysed at the mRNA level in four human UM cell lines with different metastatic potentials. BRMS1 mRNA was present in the four cell lines and their relative expression was similar. This differs from what has been published regarding BRMS1 mRNA levels in other cancers. In human breast carcinoma cell lines, for example, it was shown that the amount of BRMS1 mRNA present in the cell lines is inversely correlated with their metastatic potential [20]. Seraj *et al* [26], in a publication about human bladder cancer cell lines, demonstrated that BRMS1 mRNA expression was higher in the poorly metastatic T24 cell line than in the T24T, the highly metastatic variant. In addition, in a third study, BRMS1 mRNA levels were measured in a panel of skin melanocyte- and melanoma-derived cell lines representing a continuum of skin melanoma stages of progression and it was found that mRNA levels decreased as the cell lines’ metastatic potential increased [25].

BRMS1 was present in the four UM cell lines as demonstrated by immunocytochemistry. Western blot analysis was used to evaluate levels of BRMS1 protein expression. The immunoblotting demonstrated a positive band at ~26.8 kDa in all cell lines, and it distinguished the amount of protein between them; MKT-BR, the least metastatic of the four, had the highest protein expression. These findings agree with the attribution made to BRMS1 of suppressing metastasis: the least metastatic cell line expresses more BRMS1 than the more metastatic ones. In human breast carcinoma cell lines, it has been shown that the degree of metastasis suppression generally correlates with the level of BRMS1 protein expression [20]. However, although the mRNA and protein expression findings are interesting, mRNA and protein levels for BRMS1 do not always correlate, at least in cutaneous melanoma [54].

The mean age at the time of primary tumour diagnosis of our UM patients was 61 years, which is in agreement with the findings of other studies [9, 14, 16]. Almost 80% of our specimens stained positive for BRMS1. In accordance with what Hicks *et al* [34] demonstrated, our positive control, a breast cancer specimen, revealed nuclear staining. Interestingly, our results in UM differ from those of the latter study in that 87.50% of our specimens showed cytoplasmatic staining.

Seraj *et al* [20] investigated the predicted amino acid sequence of BRMS1 and found that it included two presumed nuclear localisation sequences and a potential endoplasmic reticulum retention sequence. Through western blots of cytosolic and nuclear fractions of 901-tagged BRMS1-transfected MDA-MB-435 cells and micrographs of phase staining and immunofluorescence, another study confirmed the predominant nuclear localisation of BRMS1 in breast carcinoma cell lines. Interestingly, the same study showed that BRMS1 restores gap junctions, meaning that it also functions at the cell surface, a role not predicted by the analysis of its protein structure [35]. In support of this cell surface function of BRMS1, a recent study by Khotskaya *et al* [56] correlated the expression of BRMS1 in breast cancer cells with a decreased ability to respond to microenvironmental changes due to a failure to reorganise their cytoskeleton resulting in a delay in cellular adhesion.

Seraj *et al* [20] further concluded that the predicted BRMS1 amino acid sequence suggests that BRMS1 interacts with other proteins, and recently Liu *et al* [55] found BRMS1 also functions as a metastases suppressor in lung cancer through an E3 ligase function on the histone acetyltransferase p300. Moreover, nuclear factor-κB (NF-κB), a transcription factor composed of p50 and p65 subunits, is sequestered in the cytoplasm through its cytosolic interaction with inhibitors of NF-κB (IκBα). Following certain stimuli, a cascade of events leads to the phosphorylation of IκBα and its subsequent degradation. This in turn leads to the liberation of NF-κB and its translocation to the nucleus where it transactivates NF-κB-responsive genes [36, 37]. NF-κB is activated in many cancers; it plays a critical role in deregulation of cell growth, angiogenesis, and tendency of tumours to metastasize [38–40], acting as one of the major signalling pathways responsible for cancer cell invasion and possibly functioning as a target for cancer therapy [41]. Using tissue microarray technology and immunohistochemistry, Li *et al* [50] demonstrated in cutaneous melanoma that via suppression of NF-κB activity as well as IL-6, BRMS1 overexpression inhibited endothelial cell growth and tube formation ability. BRMS1 seems to suppress NF-κB activity by blocking IκBα phosphorylation and degradation [51]. It seems that the downregulation of NF-κB-dependent metastasis-related gene expression is one of the ways by which BRMS1 regulates metastatic potential [42]. These findings suggest that at least one of BRMS1’s mechanisms of action is related to its cytoplasmatic localisation. Recently, Slipicevic *et al* showed a differential expression of BRMS1 in the nucleus and cytoplasm of cutaneous melanoma cells. The authors hypothesised that cytoplasmic BRMS1 restricts melanoma progression while possibly nuclear BRMS1 promotes melanoma cell invasion [52]. BRMS1 has been traditionally considered to be a nuclear transcription co-repressor due to the presence of definitive nuclear localisation signals [53]. Although cytoplasmic activities of BRMS1 warrant further investigation, it seems that the intracellular localisation determines its *in vivo* effect, which could possibly explain our cytoplasmatic staining in all of the cell lines and in 87.50% of the UM specimens. It is important to note that, like many other proteins, BRMS1 may have different roles in cutaneous and UM [57]. Further studies in UM will help to clarify the significance of subcellular localisation of BRMS1 in this particular tumour.

In UM, tumour-infiltrating macrophages are common and are associated with increased melanoma-specific mortality [43]. It was interesting to see that, in our samples, tumour infiltrating macrophages stained with anti-BRMS1 and their staining intensity was generally higher than in UM cells. Most of the studies that have been performed using immunostaining to investigate MSGs in different cancers do not mention if macrophages and other stromal cells stained for the particular protein. In contrast, Schmid *et al* [44], studying KISS-1 in hepatocellular carcinoma, found no staining in inflammatory cells, including macrophages. On the other hand, it has already been shown that *KAI-1*, another MSG, is so highly expressed in macrophages [45] that at least one study has already used it as a positive control [46]. We cannot exclude the possibility that the macrophages are staining because of cross reaction. As some authors have proposed, macrophages constitute a non-neoplastic cell population that is a potential source for cross reaction in melanomas studied by immunohistochemical methods [47, 48].

The occurrence of metastasis was similar between groups 1 and 2. This differs from what would be expected from a gene that suppresses metastasis. The expression of NM23 in UM, for example, has shown to be inversely correlated with the development of liver metastasis in nude mice [21]. No statistically significant correlation could be established between BRMS1 protein expression in UM and survival. It differs from the study involving BRMS1 immunoexpression in breast cancer, which showed that the loss of BRMS1 protein expression predicts reduced disease-free survival. Hicks *et al* [34] also found an association between BRMS1 protein expression and some prognostic factors for breast cancer, while no correlation could be established with others. In cutaneous melanoma, Li *et al* [50] demonstrated that BRMS1 expression was significantly decreased in melanoma metastases when compared with primary melanomas, and reduced BRMS1 staining was associated with worse patient survival. In our study, no statistically significant correlation could be made between BRMS1 protein expression and tumour cell type, largest tumour dimension, or tumour location, suggesting that BRMS1 does not suppress metastasis nor is it associated with prognosis in UM. Due to the small sample size in our study, we have to be cautious when interpreting or extrapolating these results.

## Conclusions

This is the first study to assess the MSG BRMS1 in UM cell lines and patient specimens; BRMS1 is expressed in UM at both the mRNA and protein levels. *In vitro* studies showed that the least metastatic cell line had higher levels of BRMS1. However, when patient samples were analysed, no statistically significant correlation could be established between BRMS1 protein expression and survival, tumour cell type, largest tumour dimension, and tumour location. More studies are needed to further dissect the roles BRMS1 has in UM.

## Figures and Tables

**Table 1.** Age, tumour location, classification, and dimension in each group.

	Group 1	Group 2
Age^*^	61.42 ± 12.88	60.14 ± 12.54
*Tumour Classification*		
Spindle-cell type Mixed-cell type	5	2
	19	5
*Tumour Location*		
Choroidal Ciliary body	17	7
	7	0

Tumour dimension^**^	13.54 ± 4.83	15.13 ± 4.42

* Mean age expressed in years.

** Mean largest tumour dimension expressed in millimetres.
